# Оценка стероидного профиля в слюне у пациентов с врожденной дисфункцией коры надпочечников

**DOI:** 10.14341/probl13328

**Published:** 2024-01-24

**Authors:** М. А. Тюльпаков, Е. В. Нагаева, Н. Ю. Калинченко, О. Б. Безлепкина

**Affiliations:** Национальный медицинский исследовательский центр эндокринологии; Национальный медицинский исследовательский центр эндокринологии; Национальный медицинский исследовательский центр эндокринологии; Национальный медицинский исследовательский центр эндокринологии

**Keywords:** врожденная дисфункция коры надпочечников, слюна, высокоэффективная жидкостная хроматография и тандемная масс-спектрометрия, 17-гидроксипрогестерон, андростендион, тестостерон

## Abstract

Врожденная дисфункция коры надпочечников (ВДКН) — группа аутосомно-рецессивных заболеваний, требующих пожизненной заместительной терапии глюкокортикоидами (ГК). Недостаточность терапии ГК приводит к преждевременному половому развитию у мальчиков, гетеросексуальному развитию у девочек, ускоренному костному созреванию и низкому конечному росту. В подростковом возрасте недостаточность терапии ГК является причиной нарушения становления менструального цикла у девочек и развития опухолевых образований яичек у мальчиков, в конечном счете — снижения репродуктивного потенциала у лиц обоих полов. С другой стороны, передозировка ГК приводит к медикаментозному синдрому Иценко–Кушинга. С целью подбора адекватных доз ГК в детском и подростковом возрасте необходимы многократные определения концентраций 17-гидроксипрогестерона, андростендиона и тестостерона плазмы крови, а следовательно — многократные заборы венозной крови. Процедура забора крови зачастую вызывает «стрессорную реакцию» у маленьких пациентов, влияя на полученные гормональные результаты, требует специально обученного медицинского персонала. В связи с этим разработка и внедрение неинвазивного метода определения стероидного профиля чрезвычайно важны при мониторинге терапии ГК у детей. Помимо этого, используемый в настоящее время иммунофлюоресцентный анализ не позволяет исследовать другие надпочечниковые стероиды, обладает высокой погрешностью за счет «перекрестной реакции» близких по строению стероидов, что завышает полученные результаты. В отличие от иммуноферментного анализа, метод жидкостной хроматографии и тандемной масс-спектрометрии более предпочтителен, так как обладает большей специфичностью и точностью. Представленный в литературном обзоре неинвазивный метод определения стероидного профиля в слюне, предлагаемой в качестве альтернативного субстрата, способен нивелировать вышеназванные недостатки, упростить и сделать более точным подбор терапии ГК у пациентов с ВДКН, что особенно важно в детском возрасте.

## Abstract (en)

Congenital adrenal hyperplasia (CAH) is a group of autosomal recessive disorders requiring lifelong glucocorticoid replacement (GC) therapy. Lack of GC therapy leads to precocious puberty in boys, heterosexual development in girls, accelerated bone maturation and short final height in both sexes. In adolescence, the lack of GC therapy is the cause of menstrual disorders in girls and the development of TART in boys, as a result reducing the reproductive potential in both sexes. On the other hand, an overdose of GC leads to drug-induced Itsenko-Cushing’s syndrome. In order to select adequate doses of GC in childhood and adolescence, multiple determinations of 17-hydroxyprogesterone, androstenedione, and testosterone in blood plasma, and thus multiple venous blood sampling are required. The blood sampling requires specially trained medical staff and can effect on the results due to stress reaction especially in young patients. Hence, the development and implementation of a non-invasive method for determining the steroid profile is extremely important in monitoring GC therapy in children. In addition, the currently used immunofluorescence assay cannot determine other adrenal steroids, has a high variation due to the «cross-reaction» of steroids that are similar in structure, which inflates the results. Unlike immunofluorescence assay, liquid chromatography and tandem mass spectrometry is more preferable method, since it is more specific and accurate. In this literature review, saliva presented as an alternative substrate and the non-invasive method for determining the steroid profile. This method can solve the above disadvantages, simplify and make more accurate the selection of GC therapy in patients with CAH, which is especially important in childhood.

## ВВЕДЕНИЕ

Врожденная дисфункция коры надпочечников (ВДКН) — это группа аутосомно-рецессивных заболеваний, в основе которых лежит дефект одного из ферментов или транспортных белков, принимающих участие в биосинтезе кортизола в коре надпочечников. Наиболее частой причиной ВДКН является дефицит 21-гидроксилазы, с первых недель после рождения и на протяжении всей жизни требующий замещения глюкокортикоидами (ГК) [1–4]. Недостаточность терапии ГК приводит к преждевременному половому развитию у мальчиков, гетеросексуальному развитию у девочек, ускоренному костному созреванию, быстрому закрытию зон роста и низкому конечному росту. В подростковом возрасте недостаточность терапии ГК является причиной нарушения становления менструального цикла у девочек, развития опухолевых образований яичек у мальчиков, в конечном счете — снижения репродуктивного потенциала у лиц обоих полов. С другой стороны, передозировка ГК сопровождается ожирением, низкорослостью, артериальной гипертензией, развитием метаболического синдрома в более старшем возрасте [5–7]. В связи с этим подбор доз ГК в детском возрасте крайне важен.

Общепринятыми маркерами оценки эффективности терапии ВДКН являются: 17-ОН-прогестерон (17ОНП), андростендион и тестостерон плазмы крови, измеренные методом иммунофлюоресцентного анализа (ИФА) [1–4]. Однако данные методики не являются оптимальными, так как, с одной стороны, не позволяют оценить другие стероидные маркеры заболевания, а с другой — обладают высокой степенью погрешности, завышением полученных результатов за счет так называемой «перекрестной реакции» близких по строению стероидов [8–10].

В последнее время для исследования стероидного профиля в плазме используется метод высокоэффективной жидкостной хроматографии и тандемной масс-спектрометрии (ВЭЖХ МС/МС), так как, в отличие от ИФА, ВЭЖХ МС/МС позволяет исследовать одновременно концентрации нескольких надпочечниковых стероидов с высокой точностью [11–14]. Помимо этого, при интерпретации полученных результатов необходимо учитывать тот факт, что забор крови является «стрессорным» фактором для ребенка и может приводить к повышению уровней исследуемых стероидов [15]. В этой связи использование слюны в качестве субстрата для определения стероидов представляется перспективным неинвазивным методом.

## ОБРАЗОВАНИЕ СЛЮНЫ И ПОПАДАНИЕ В НЕЕ СТЕРОИДОВ

В железистых клетках ацинусов слюнных желез находятся секреторные гранулы, в которых осуществляется синтез ферментов и муцина. Образующийся первичный секрет из клеток попадает в протоки, где он разбавляется водой и насыщается минеральными веществами. Околоушные железы в основном образованы серозными клетками и вырабатывают жидкий серозный секрет, в то время как подъязычные — слизистыми, которые выделяют слюну, богатую муцином. Подчелюстные — вырабатывают смешанную серозно-слизистую слюну [16].

Существует два основных механизма попадания стероидных гормонов в слюну.

Первый — внутриклеточная пассивная диффузия, посредством которой жирорастворимые «несвязанные» стероиды могут проходить сквозь клеточную мембрану ацинусов слюнных желез. Их концентрация не зависит от скорости секреции слюны, поэтому определяемый уровень гормонов в слюне совпадает с таковым в ­крови [17][18].

Второй — ультрафильтрация. «Связанные» с белками стероиды крови вместе с водой проникают через плотные перегородки в ацинусы. Их концентрация сильно зависит от скорости потока, поэтому определяемый уровень стероидов в слюне значительно ниже, чем в ­крови [17][18].

## МЕТОДЫ СБОРА СЛЮНЫ

В настоящее время существует несколько методов сбора слюны, однако стандартизированного варианта не существует, так как каждый из них так или иначе влияет на анализируемые стероиды.

## ПАССИВНОЕ СЛЮНОТЕЧЕНИЕ

С 1934 г. «золотым стандартом» сбора слюны считается пассивное слюнотечение, поскольку оно позволяет исключить влияние скорости потока слюны на ее состав. В емкости для сбора (например полипропиленовые ­эппендорфы) пациенту необходимо, не прилагая активных действий, позволить стечь слюне.

В 2007 г. D. Granger и соавт. отметили преимущества пассивного слюнотечения, такие как возможность собрать большой объем биоматериала и малое влияние внешних факторов, имеющих место при других методиках, или стимуляции слюноотделения. Однако у этого метода также есть свои ограничения: он неприменим у пациентов, которым невозможно объяснить принцип сбора (например, маленький ребенок, пожилой человек и др.) [19].

## ВЫПЛЕВЫВАНИЕ СЛЮНЫ

Выплевывание слюны — это метод сбора слюны, при котором человек сначала собирает слюну на дне ротовой полости, а затем выплевывает собранное в заранее подготовленный контейнер. Данный метод быстрее предыдущего, однако он тоже имеет ограничения, а за счет активного процесса слюна становится «стимулиро­ванной» [20].

## СБОР СЛЮНЫ С ПОМОЩЬЮ ТАМПОНА

Слюна может быть собрана с помощью тампона из хлопка или других синтетических материалов, помещенного в ротовую полость. От выбора материала зависит точность определяемых аналитов (материал может абсорбировать на себя ту или иную часть интересующего аналита). В литературе наиболее часто упоминаемыми устройствами для сбора слюны являются Salivette (Sarstedt) и SalivaBio Oral Swab (SOS, Salimetrics). Salivette выпускаются в 3 вариантах: с полипропиленовым, ­полиэтиленовым легкой плотности и хлопковым тампоном. Все варианты производятся одинаковой формы и размеров. Основной отличительной чертой является цвет крышки устройства: белая крышка говорит о хлопковом тампоне; синяя/голубая — о синтетическом. Для сбора слюны пациенту необходимо поместить тампон в ротовую полость до полного пропитывания слюной, данный процесс может занимать до 10 минут. Необходимо избегать непроизвольного проглатывания тампона (что может быть важным при сборе образцов у детей) [17].

## ОПРЕДЕЛЕНИЕ СТЕРОИДОВ В СЛЮНЕ

Одно из первых исследований в данной области было проведено еще в 70-х годах прошлого века. R. Walker и соавт. (1979) проводили сравнительный анализ содержания 17ОНП в слюне и крови у пациентов от 1 до 16 лет с классической 21-гидроксилазной недостаточностью, находящихся на лечении пероральным гидрокортизоном в дозе 15–20 мг/м²/сут и флудрокортизоном 0,1–0,15 мг/м²/сут, а также у 32 здоровых добровольцев методом радиоиммунного анализа (РИА). Сбор образцов крови и слюны производился одномоментно в промежутке времени с 9 до 10 ч утра. Слюна собиралась непосредственно в стеклянные пробирки. У детей раннего возраста слюна собиралась с помощью шприца со дна полости рта. В результате было установлено, что у контрольной группы среднее содержание 17ОНП в слюне соответствовало 390 пмоль/л (от 90 до 1520 пмоль/л). Диапазон концентраций 17ОНП в основной группе варьировал от 67 до 26300 пмоль/л [21].

Возможности использования слюны для анализа стероидов продемонстрировала О. Фофанова (1989) в своей диссертации, защищенной на базе Института клинической эндокринологии Всесоюзного эндокринологического центра Академии медицинских наук СССР. В этом исследовании участвовали 30 детей допубертатного возраста, от 3 до 11 лет, с классической 21-гидроксилазной недостаточностью (вирильной формой) и 33 условно здоровых ребенка. Все дети были разделены на две группы: 1-я — с допубертатным костным возрастом, 2-я — с пубертатным. Парные пробы крови и слюны собирались исследователем 6 раз в течение суток: в 8:00, 12:00, 16:00, 20:00, 24:00, 4:00. Забор слюны предшествовал забору крови. Пробы смешанной цельной слюны (по 4–6 мл) собирались детьми в центрифужную стеклянную пробирку. Методом РИА была выявлена сильная положительная корреляция между концентрациями тестостерона в обеих биологических жидкостях (r=0,766; р<0,0001). Сильная положительная корреляция была получена и при исследовании 17ОНП в парных пробах сыворотки и слюны (r=0,818; р<0,0001). Средние анализируемые концентрации стероидов в крови и слюне представлены в таблице 1.

**Table:** Таблица 1. Средние анализируемые концентрации стероидов в крови и слюне

Тестостерон, нмоль/л	17-гидроксипрогестерон, нг/мл
Общая группа	1 группа	2 группа	1 группа	2 группа
Кровь	Слюна	Кровь	Слюна	Кровь	Слюна	Кровь	Слюна	Кровь	Слюна
3,94±0,23	0,192±0,011	2,41±0,19	0,142±0,014	4,95±0,32	0,218±0,015	131,5±13,2	2,96±0,40	120,3±9,6	3,87±0,41

Повторное исследование вышеуказанных показателей было проведено через 6 мес после коррекции терапии ГК. Отмечалась положительная динамика в виде снижения выраженности гиперандрогении и средней концентрации тестостерона и 17ОНП в крови и слюне. Результаты повторных исследований представлены в таблице 2.

**Table:** Таблица 2. Средние анализируемые концентрации стероидов в крови и слюне при повторном обследовании через 6 месяцев после проведенной коррекции терапии ГК

Тестостерон, нмоль/л	17-гидроксипрогестерон, нг/мл
Общая группа	2 группа
Кровь	Слюна	Кровь	Слюна
1,48±0,18	0,075±0,049	8,8±1,04	0,61±0,11

Автор допустил использование слюны не только в диагностическом процессе, но также и для контроля лечения этого заболевания [22].

N. Shindo и соавт. (1990) определили наличие 8 стероидных гормонов: тестостерон, 17ОНП, 17ОН-прегненолон, прогестерон, прегненолон, 11-дезоксикортизол, кортизол и альдостерон в слюне методом жидкостной хроматографии/масс-спектрометрии с термоспреем у двух пациентов с ВДКН, получающих заместительную гормональную терапию, и у здорового добровольца. В своей работе авторы не указали, какой метод сбора слюны они использовали, а также не проводили количественный анализ [23].

M. Groshl и соавт. (2003) разработали референсные значения для кортизола, 17ОНП и прогестерона, исследовав данные параметры в слюне у 252 детей в возрасте от 4 дней до 15 лет. Сбор слюны проводился в 7:00, 13:00 и 19:00. Для этого использовались специальные наборы для сбора слюны Salivette с тампонами из полиэстера (компания-производитель Sarstedt) или для новорожденных детей с помощью модифицированной пустышки. В соске пустышки были проделаны отверстия, а полоски фильтровальной бумаги, используемой в карточках для проведения анализа методом сухих пятен крови, были помещены внутрь соски. Абсорбирующий материал не влиял на полученные результаты методом РИА. Были разработаны референсные значения для групп ­детей старше 4 нед, 1–12 мес, 1–2 лет и 2–15 лет. Полученные результаты представлены в таблице 3.

**Table:** Таблица 3. Референсные значения для кортизола, 17ОНП и прогестерона в слюне для разных возрастных групп

	Возраст
<4 нед (n=13)	1–12 мес	1–2 года	2–15 лет
Кортизол, нмоль/л	Утро	Среднее значение (отклонение)	34,5 (8,5)	24,3 (12,0)	15,0 (10,8)	24,7 (8,5)
	Диапазон	20,4–48,3	11,3–50,8	5,8–42,2	3,0–54,9
День	Среднее значение (отклонение)	30,5 (9,6)	11,5 (8,9)	6,2 (2,5)	8,0 (4,0)
	Диапазон	11,3–44,1	3,3–30,9	2,2–9,7	1,1–20,7
Вечер	Среднее значение (отклонение)	27,5 (10,9)	3,8 (3,0)	1,6 (1,0)	1,7 (1,4)
	Диапазон	3,3–43,3	0,6–9,4	0,3–3,0	0,2–8,7
17ОНП, пмоль/л	Утро	Среднее значение (отклонение)	306 (75)	130 (41)	125 (41)	85 (49)
	Диапазон	168–402	84–198	63–195	6–225
День	Среднее значение (отклонение)	322 (94)	112 (25)	67 (27)	58 (36)
	Диапазон	123–450	84–144	24–105	3–195
Вечер	Среднее значение (отклонение)	163 (38)	63 (21)	40 (21)	27 (19)
	Диапазон	90–216	36–93	15–66	3–93
Прогестерон, пмоль/л	Утро	Среднее значение (отклонение)	677 (305)	235 (138)	115 (41)	137 (81)
	Диапазон	350–1320	89–525	60–169	29–601
День	Среднее значение (отклонение)	423 (240)	102 (38)	63 (21)	101 (53)
	Диапазон	153–875	51–162	25–99	16–282
Вечер	Среднее значение (отклонение)	381 (191)	122 (67)	52 (24)	77 (46)
	Диапазон	80–716	35–239	25–86	11–267

Для детей старше 1 года различий в определяемых аналитах в зависимости от пола выявлено не было (p=0,05) [24].

A.Z. Juniarto и соавт. (2011) проводили сравнительный анализ содержания 17ОНП и андростендиона в крови и слюне методом ИФА. С этой целью у 24 пациентов с ВДКН в возрасте от 1,4 до 16,3 года собирали слюну с помощью специальных наборов для сбора слюны Salivette с тампонами из полиэстера (компания-производитель Sarstedt). Все пациенты получали заместительную терапию гидрокортизоном 12 мг/м²/сут, разделенную на 3 приема. Сбор слюны и крови предшествовал приему гидрокортизона. Исследователи получили положительную корреляцию содержания 17ОНП (R=0,929, p<0,01) и андростендиона (R=0,611, p<0,01) в слюне и крови [25].

Большой вклад внесла Ж.Е. Белая (2013), подтвердив в своей диссертации, защищенной на базе ФГБУ «Эндокринологический научный центр» МЗ РФ, возможность использования слюны как альтернативного биоматериала для выявления гиперсекреции кортизола. В исследовании образцы были взяты у 255 человек (98 здоровых добровольцев с нормальным индексом массы тела и 127 пациентов с ожирением, в том числе 10 человек с избыточной массой тела, которые имели клинические признаки эндогенного гиперкортицизма). Сбор образцов проводился в специальные наборы для сбора слюны Salivette с тампонами из полиэстера (компания производитель Sarstedt). В результате исследования содержания свободного кортизола слюны, собранной в 23:00, был разработан референтный интервал (минимальный 0,5 нмоль/л, максимальный 14,5 нмоль/л) для здоровых добровольцев. Воспроизводимость определения свободного кортизола в вечерней слюне оценивалась при сравнении измерения уровня кортизола в двух образцах слюны. Всего два образца сдали 205 человек (410 образцов), статистически значимых различий между двумя тестами выявлено не было (р=0,12). Коэффициент корреляции между двумя образцами составил R=0,785 (р<0,0001). Полученные в данном исследовании результаты используются для подтверждения эндогенного гиперкортицизма у детей и взрослых по сей день с минимальной погрешностью [26].

Е. Jurgens и соавт. (2019) занимались одномоментным количественным определением 7 стероидов (андростендион, кортизон, кортизол, 11-дезоксикортизол, 21-дезоксикортизол, 17ОНП и тестостерон) в слюне с помощью ВЭЖХ МС/МС. Для этого исследования образцы собирались у 19 «здоровых» добровольцев младше 20 лет. Был выбран метод «прямого выплевывания слюны» в полипропиленовые ампулы. Основываясь на полученных результатах, были определены минимальная и максимальная концентрация для кортизола, кортизона, тестостерона и андростендиона. Полученные результаты представлены в таблице 4. Не было никаких существенных различий в зависимости от пола в вышеперечисленных аналитах, за исключением тестостерона, который у мужчин был ожидаемо выше (p<0,05). В исследуемых образцах не удалось обнаружить 21-дезоксикортизол, 11-дезоксикортизол и 17ОНП. только в нескольких образах их удалось определить в крайне низких концентрациях [27].

**Table:** Таблица 4. Диапазон концентраций стероидов в слюне у 19 «здоровых» добровольцев младше 20 лет

Нг/мл	Андростендион	Кортизол	Кортизон	Тестостерон
Мужчины	0,08–0,25	0,93–4,4	4,5–9,8	0,12–0,23
Женщины	0,06–0,21	1,5–5,6	5,6–14	0,05–0,11

I. Bacila и соавт. (2019) провели мультицентровое исследование, в котором проводился сравнительный анализ содержания 5 стероидов: 17ОНП, андростендиона, тестостерона, 11-гидроксиандростендиона и 11-кетотестостерона в слюне и плазме крови у пациентов с классической 21-гидроксилазной ВДКН. В исследование были включены 78 детей в возрасте от 8 до 18 лет и группа контроля, сопоставимая по возрасту, полу и индексу массы тела. Слюна собиралась методом «пассивного слюнотечения» в полипропиленовый сосуд с одномоментным сбором венозной крови. Все дети получали заместительную терапию ГК: 72 пациента — гидрокортизон в суточной дозе от 4 до 27 мг/м², 6 пациентов — преднизолон в суточной дозе от 3 до 4 мг/м² (в пересчете на гидрокортизон от 15 до 20 мг/м²). Концентрации гормонов в плазме и слюне были значительно выше у пациентов, чем в контроле (P<0,001) для 4 исследуемых стероидных гормона (17ОНП, андростендион, 11-гидроксиандростендион и 11-кетотестостерон). У пациентов с ВДКН для всех 5 гормонов была обнаружена сильная корреляция между плазмой и слюной. Наиболее положительная корреляция отмечалась для концентрации андростендиона (rs=0,931; P<0,001) и 11-кетотестостерона (rs=0,944; P<0,001). Более слабая корреляция выявлена для концентрации тестостерона (rs=0,867; P<0,001), 17ОНП (rs=0,871; P<0,001) и 11-гидроксиандростендиона (rs=0,876; P<0,001) [28].

B.P.H. Adriaansen и соавт. (2022) занялись разработкой референсных интервалов для 17ОНП и андростендиона у здоровых добровольцев. С этой целью была собрана слюна у 255 здоровых добровольцев в возрасте от 4 до 75 лет в 3 временных точках (с 7:00 до 8:00; с 15:00 до 16:00 и с 22:00 до 23:00). В выборку не вошли девочки 10–15 лет и мальчики 11–15 лет, так как активный пубертат в данных возрастных группах может влиять на итоговые показатели. Данные временные интервалы были выбраны с учетом стандартного приема заместительной терапии пациентами с ВДКН. Для сбора слюны применялся метод прямого стекания в специальный набор для сбора слюны (полипропиленовый флакон компании BD Biosciences). Анализ проводился с помощью ЖХ МС/МС. В результате были разработаны референсные значения для нескольких возрастных групп: с 4 до 10 лет с 16 до 51 лет. Полученные результаты представлены в таблице 5 [29].

**Table:** Таблица 5. Референсные интервалы для андростендиона и 17ОНП в слюне для детей 4–10 лет и для взрослых 16–51 года

Андростендион	Дети	10–123 (n=80)	<7–54 (n=77)	<7–42 (n=80)
Взрослые	119–553 (n=57)	40–363 (n=57)	42–319 (n=59)
17ОНП	Дети	<110 (n=78)	<22 (n=76)	<20 (n=78)
Взрослые	<170 (n=57)	<110 (n=56)	<67 (n=58)

## ЗАКЛЮЧЕНИЕ

Контроль стероидного профиля у детей с ВДКН крайне важен для адекватного подбора заместительной терапии. Согласно клиническим рекомендациям, протокол наблюдения детей с ВДКН подразумевает определение уровня стероидов в крови в зависимости от возраста и степени компенсации основного заболевания 1 раз в 1–6 мес. Забор крови является «стрессорным» и крайне трудозатратным, особенно у детей первого года жизни. В связи с этим поиск альтернативного пути забора биоматериала для контроля проводимой терапии крайне важен. Согласно вышеизложенным исследованиям, слюна может быть хорошей альтернативой венозной крови для контроля проводимой терапии у пациентов с классической 21-гидроксилазной недостаточностью. Вместе с тем для разработки стандартизированного метода необходимы дальнейшие исследования в данной области.

## ДОПОЛНИТЕЛЬНАЯ ИНФОРМАЦИЯ

Источники финансирования. Работа выполнена в рамках государственного задания «Персонализация терапии врожденной дисфункции коры надпочечников на основе исследования стероидного профиля слюны» №123021000036-2.

Конфликт интересов. Авторы декларируют отсутствие явных и потенциальных конфликтов интересов, связанных с содержанием настоящей статьи.

Участие авторов. Все авторы одобрили финальную версию статьи перед публикацией, выразили согласие нести ответственность за все аспекты работы, подразумевающую надлежащее изучение и решение вопросов, связанных с точностью или добросовестностью любой части работы.
